# Aggressive variants of prostate cancer: underlying mechanisms of neuroendocrine transdifferentiation

**DOI:** 10.1186/s13046-022-02255-y

**Published:** 2022-02-02

**Authors:** Lina Merkens, Verena Sailer, Davor Lessel, Ella Janzen, Sarah Greimeier, Jutta Kirfel, Sven Perner, Klaus Pantel, Stefan Werner, Gunhild von Amsberg

**Affiliations:** 1 Department of Tumor Biology, University Medical Center Hamburg-Eppendorf, Martinistrasse 52, 20246 Hamburg, Germany; 2 Institute of Pathology, University of Luebeck and University Hospital Schleswig-Holstein, Campus Luebeck, Ratzeburger Allee 160, 23538 Luebeck, Germany; 3 Institute of Human Genetics, University Medical Center Hamburg-Eppendorf, Martinistrasse 52, 20246 Hamburg, Germany; 4 Pathology, Research Center Borstel, Leibniz Lung Center, Borstel, Germany; 5 European Liquid Biopsy Society (ELBS), Hamburg, Germany; 6 Mildred Scheel Cancer Career Center Hamburg HaTRiCs4, University Cancer Center Hamburg, University Medical Center Hamburg-Eppendorf, Hamburg, Germany; 7 Department of Hematology and Oncology, University Cancer Center Hamburg, University Medical Center Hamburg-Eppendorf, Martinistrasse 52, 20246 Hamburg, Germany; 8 Martini-Klinik, Prostate Cancer Center, University Medical Center Hamburg-Eppendorf, Martinistrasse 52, 20246 Hamburg, Germany

**Keywords:** Aggressive variant prostate cancer, Small cell prostate cancer, Neuroendocrine prostate cancer, Neuroendocrine transdifferentiation

## Abstract

Prostate cancer is a hormone-driven disease and its tumor cell growth highly relies on increased androgen receptor (AR) signaling. Therefore, targeted therapy directed against androgen synthesis or AR activation is broadly used and continually improved. However, a subset of patients eventually progresses to castration-resistant disease. To date, various mechanisms of resistance have been identified including the development of AR-independent aggressive variant prostate cancer based on neuroendocrine transdifferentiation (NED). Here, we review the highly complex processes contributing to NED. Genetic, epigenetic, transcriptional aberrations and posttranscriptional modifications are highlighted and the potential interplay of the different factors is discussed.

**Background**

Aggressive variant prostate cancer (AVPC) with traits of neuroendocrine differentiation emerges in a rising number of patients in recent years. Among others, advanced therapies targeting the androgen receptor axis have been considered causative for this development. Cell growth of AVPC often occurs completely independent of the androgen receptor signal transduction pathway and cells have mostly lost the typical cellular features of prostate adenocarcinoma. This complicates both diagnosis and treatment of this very aggressive disease. We believe that a deeper understanding of the complex molecular pathological mechanisms contributing to transdifferentiation will help to improve diagnostic procedures and develop effective treatment strategies. Indeed, in recent years, many scientists have made important contributions to unravel possible causes and mechanisms in the context of neuroendocrine transdifferentiation. However, the complexity of the diverse molecular pathways has not been captured completely, yet. This narrative review comprehensively highlights the individual steps of neuroendocrine transdifferentiation and makes an important contribution in bringing together the results found so far.

## Background

Prostate cancer (PCa) is primarily a hormone-driven disease mediated by androgen receptor (AR) signaling-driven cell growth. Elevated serum concentrations of the AR downstream target prostate specific antigen (PSA) are indicative of this AR-mediated tumor growth. Androgen deprivation therapy (ADT) with Gonadotropin Releasing Hormone agonists or antagonists is the backbone of treatment for advanced hormone sensitive PCa. However, acquisition of resistance mechanisms that restore androgen supply or AR activity in the tumor such as AR amplifications, mutations or splice variants eventually can result in castration resistant prostate cancer (CRPC) [1]. New hormonal agents (NHA) targeting androgen synthesis or binding such as abiraterone and enzalutamide show life prolonging activity in CRPC indicating that the AR signaling pathway still has a major impact for progression of disease. However, apart from those “AR-dependent” castration-resistant adenocarcinomas, a subset of patients has been found to progress with AR-independent cancer biology with a short-term response to hormonal treatment, early and extensive visceral metastases and poor outcomes. Of note, this aggressive variant prostate cancer (AVPC) is frequently associated with low PSA production and thus not recognized by PSA monitoring. Therefore, an early identification of progressive patients remains challenging.

Clinically, AVPC (formerly known as anaplastic prostate cancer) has been suggested to be defined by at least one of the following characteristics, formulated by Aparicio et al.: 1) Histological evidence of small cell neuroendocrine prostate cancer (NEPC); 2) presence of exclusively visceral metastases; 3) predominant lytic bone metastases; 4) bulky lymphadenopathy or bulky high-grade tumor mass in the prostate/pelvis; 5) low PSA at initial presentation plus high volume bone metastases; 6) presence of neuroendocrine markers on histology or serum at initial diagnosis or progression, plus any of: elevated serum lactate dehydrogenase, malignant hypercalcemia and/or elevated serum carcinoembryonic antigen in the absence of other features; 7) short interval (≤ 6 m) to androgen-independent progression following the initiation of hormonal therapy with or without the presence of neuroendocrine markers [2].

## Histopathology

**Fig. 1:**
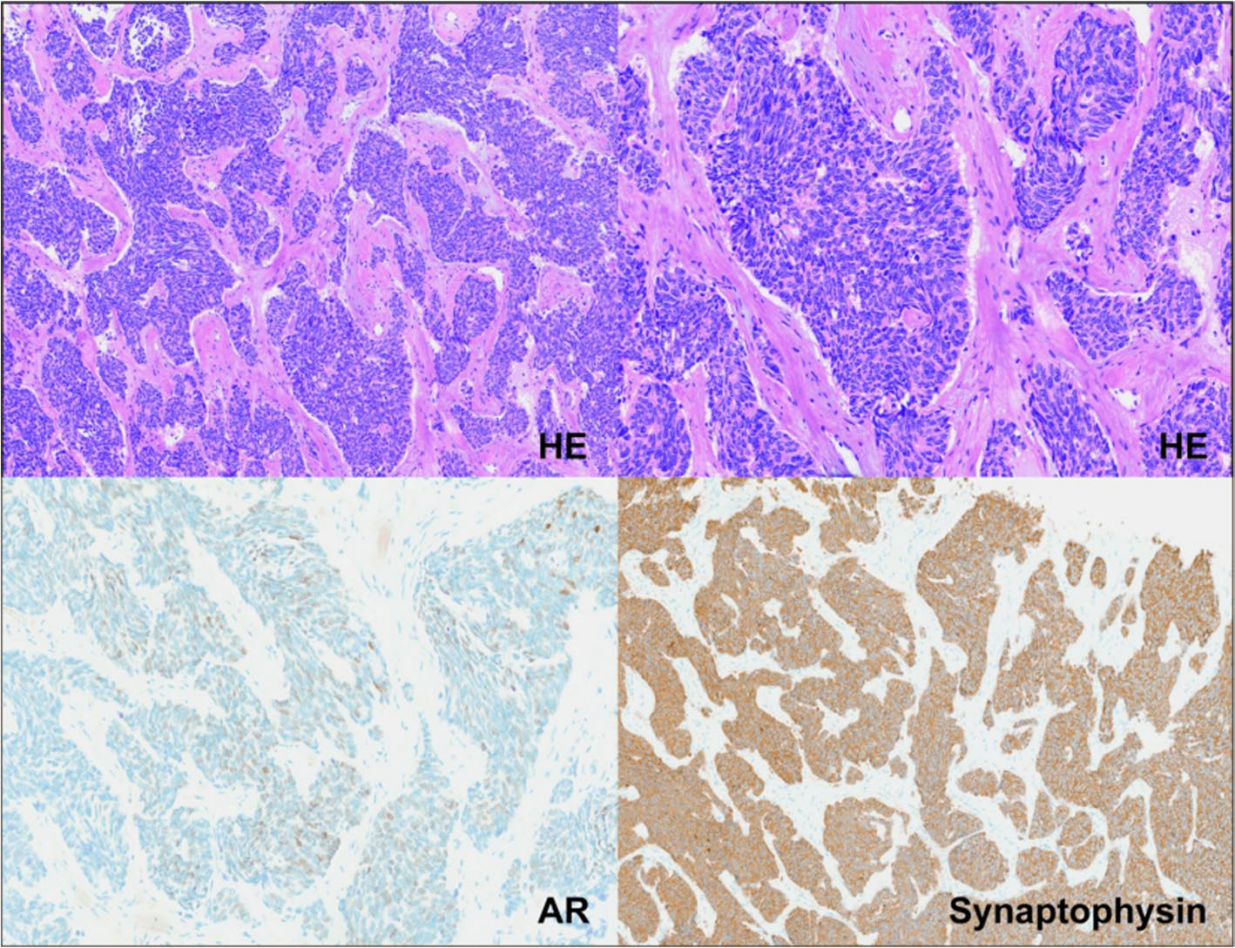
Histologic staining of small-cell NEPC. **Top** Small cell neuroendocrine prostate cancer with typical features such as scant cytoplasm, granular chromatin and a high number of apoptoses and mitoses, magnification 10x (**left**), 20 x (**right**), **Lower left** Incomplete loss of AR expression, magnification 20x, **Lower right** expression of neuroendocrine marker synaptophysin, magnification 10x

Histopathological and molecular features of AVPC vary on an inter- and intra-tumoral level indicating a heterogeneous disease. AVPC may present as small cell carcinoma displaying the typical morphology of tumor cells with scant or no cytoplasma, lack of nucleoli and crush artefact [3]. These tumors usually express neuroendocrine (NE) markers as detected by immunohistochemistry including chromogranin A (CHGA), synaptophysin (SYP), neuron-specific enolase 2 (ENO2) and neural cell adhesion molecule 1 (NCAM1, CD56). Expression of PSA and AR is often lost. Representative histopathologic images are shown in Fig. 1. In addition to pure small cell carcinoma, tumors with mixed histology consisting of high-grade adenocarcinoma and a small cell (or large cell) neuroendocrine component have been described in the AVPC category [4]. Usual high-grade adenocarcinomas can also exhibit expression of neuroendocrine markers. However, NE expression in these cases is not an adverse prognostic marker and these tumors are distinctly different from AVPC [5]. A small subset of AVPC are tumors that neither express AR nor NE markers and are thus termed “double-negative” [6, 7].

## Terminology

Nomenclature of AVPC and other NE-marker expressing tumors remains challenging because clinical and molecular features do not always obviously concur. Moreover, these tumors frequently present with varying degrees of expression of AR pathway genes, neuroendocrine markers or stem cell-like states [7, 8]. Thus, they cannot easily be fitted into finite subtypes. Due to ever-growing complexity, different terms have been established and used by researchers in this field. In order to distinguish between de novo NE tumors and those that develop in response to targeted therapy, the term treatment-emergent neuroendocrine prostate carcinoma (t-NEPC) will be used throughout this review, when referring to small cell neuroendocrine or mixed tumors not apparent at first diagnosis, as this term is meanwhile frequently used in the literature [1, 8, 9]. However, the terminology was not consistent over the past years and thus may vary throughout this review. The term mCRPC is used to refer to metastatic castration-resistant adenocarcinoma of the prostate.

## Incidence of NEPC and AVPC

NEPC is rarely diagnosed de novo in its pure form (small cell or large cell prostate cancer, < 2% of first diagnoses) [10], but about 10-17% of patients with metastatic CRPC have been reported to progress with t-NEPC when treated with NHA [11]. Increased therapeutic pressure on the AR signaling pathway due to broad implementation of NHA is assumed to be causative for a rising number of t-NEPC. For example, Abida et al. described an increase of NEPC in mCRPC tissue biopsies from NHA-treated patients (10.5%) compared to NHA-naïve patients (2.3%) [12]. Along with the approval of abiraterone and enzalutamide an increased incidence of patients with NE^+^ tumors from 6.3% in the time period from 1998 to 2011 to 13.3% in 2012-2016 was reported [6]. Of note, a rising age-adjusted incidence rate of NEPC was also observed in the years 2004-2011 suggesting other factors to promote this phenomenon (e.g. increased life expectancy or therapeutic pressure by chemotherapies) [13].

To date, most data are available on NEPC while double negative tumors are still poorly understood. Therefore, this review will focus on the molecular mechanisms and signaling pathways that have been described to contribute to the development of t-NEPC. Key aberrations found in t-NEPC are introduced and interactions potentially involved in the emergence of NE features are highlighted.

## Origin of t-NEPC

**Fig. 2:**
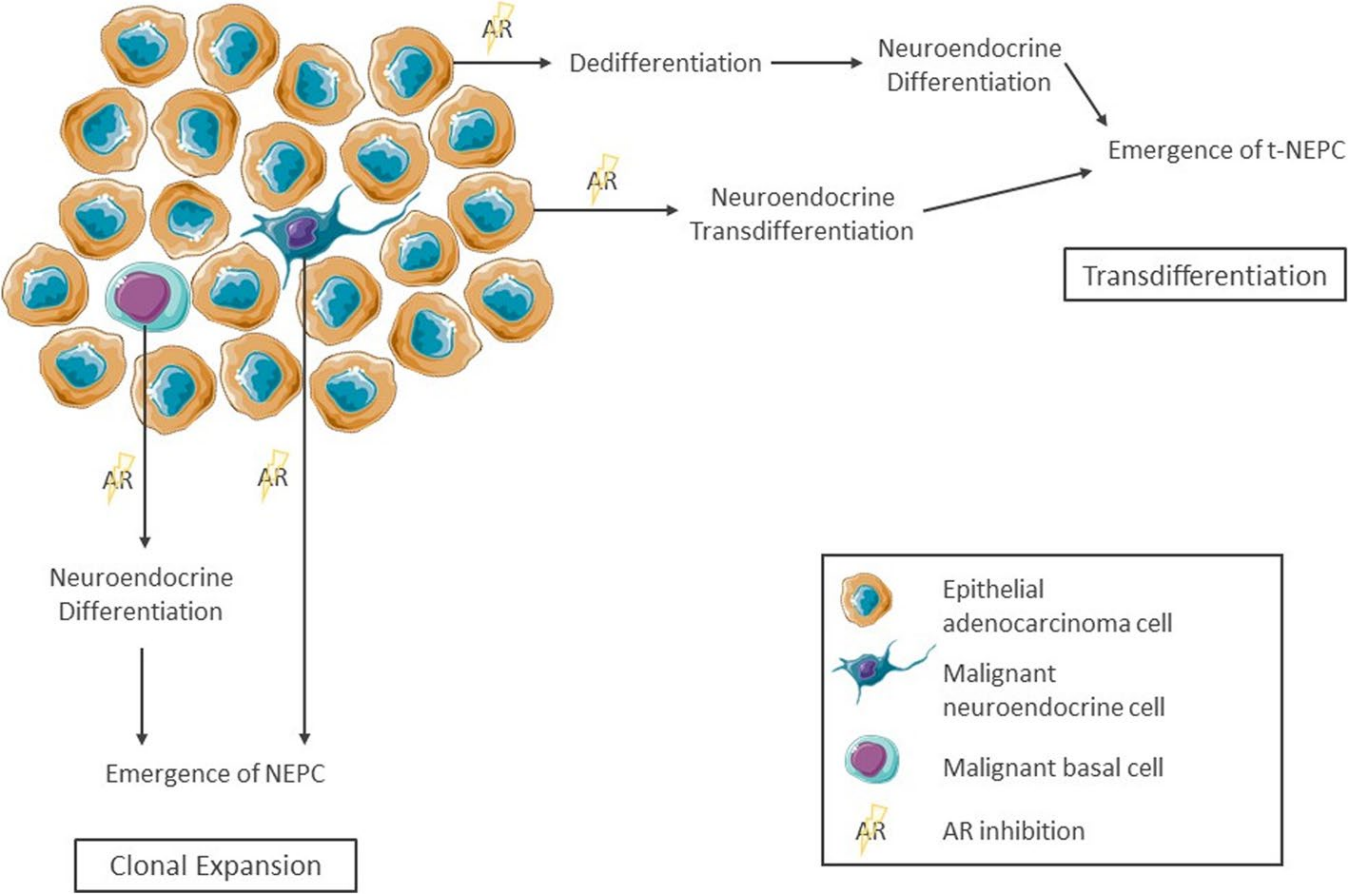
Origin of t-NEPC. Two opposing theories have been proposed to explain the origin of t-NEPC: Clonal expansion – the outgrowth of neuroendocrine or NE-differentiated basal cells – and transdifferentiation of adenocarcinoma cells

To date, there are two partially contradictory ideas on the primary cellular origin of t-NEPC - both supported by experimental evidence. Both are schematically shown in Fig. 2. The first involves clonal evolution of t-NEPC from basal or neuroendocrine cells, which are sparsely distributed in the healthy prostate [14, 15]. Before initiation of ADT and NHA, these cells remain small in number as they are outgrown by the AR-positive adenocarcinoma cells. However, as AR gets inhibited, their AR-independency is a major growth advantage resulting in development of NEPC [16]. Lee et al. recently suggested basal cells as the origin of NEPC based on findings of lineage tracing experiments in the transgenic adenocarcinoma of the mouse prostate model [14].

Second, a mechanism of transdifferentiation from epithelial adenocarcinoma cells to neuroendocrine cells has been suggested. Indicative for this process are experimental findings by Lotan et al.*,* which showed that ERG gene rearrangements are found at equal frequencies in a cohort of patients with small cell carcinoma compared to adenocarcinoma. In patients with mixed adenocarcinoma and small cell carcinoma, the ERG status was highly congruent [17, 18]. As these fusions lead to the activation of ERG in an AR-dependent manner, clones with these aberrations confer a growth advantage only in hormone-driven disease. Furthermore, copy number analyses have shown that AR amplification is similarly distributed in patients with mCRPC and those with t-NEPC [11]. In fact, the loss of AR rarely occurs due to genomic aberrations, but rather by epigenetic or posttranscriptional mechanisms [1]. Generally, point mutations and copy number aberrations have been found to be largely concurring in prostate adenocarcinoma and t-NEPC, whereas, considerable differences on transcriptional and epigenetic regulation have been described [19]. In addition, re-exposure to androgens has reversed induced NE-like transdifferentiation in LNCaP prostate adenocarcinoma cells [20]. Taken together these results strongly support the notion of a transdifferentiation mechanism driving the emergence of t-NEPC in the majority of patients. An unanswered question remains whether this process involves a dedifferentiation step in which the adenocarcinoma cells first loose AR-specific gene expression and acquire basal or stem-like properties before proceeding to a second step in which they differentiate into NE cells, or whether the transdifferentiation arises directly skipping this putative intermediate stem-like cell stage [21, 22].

## Mechanisms of t-NEPC development

**Fig. 3:**
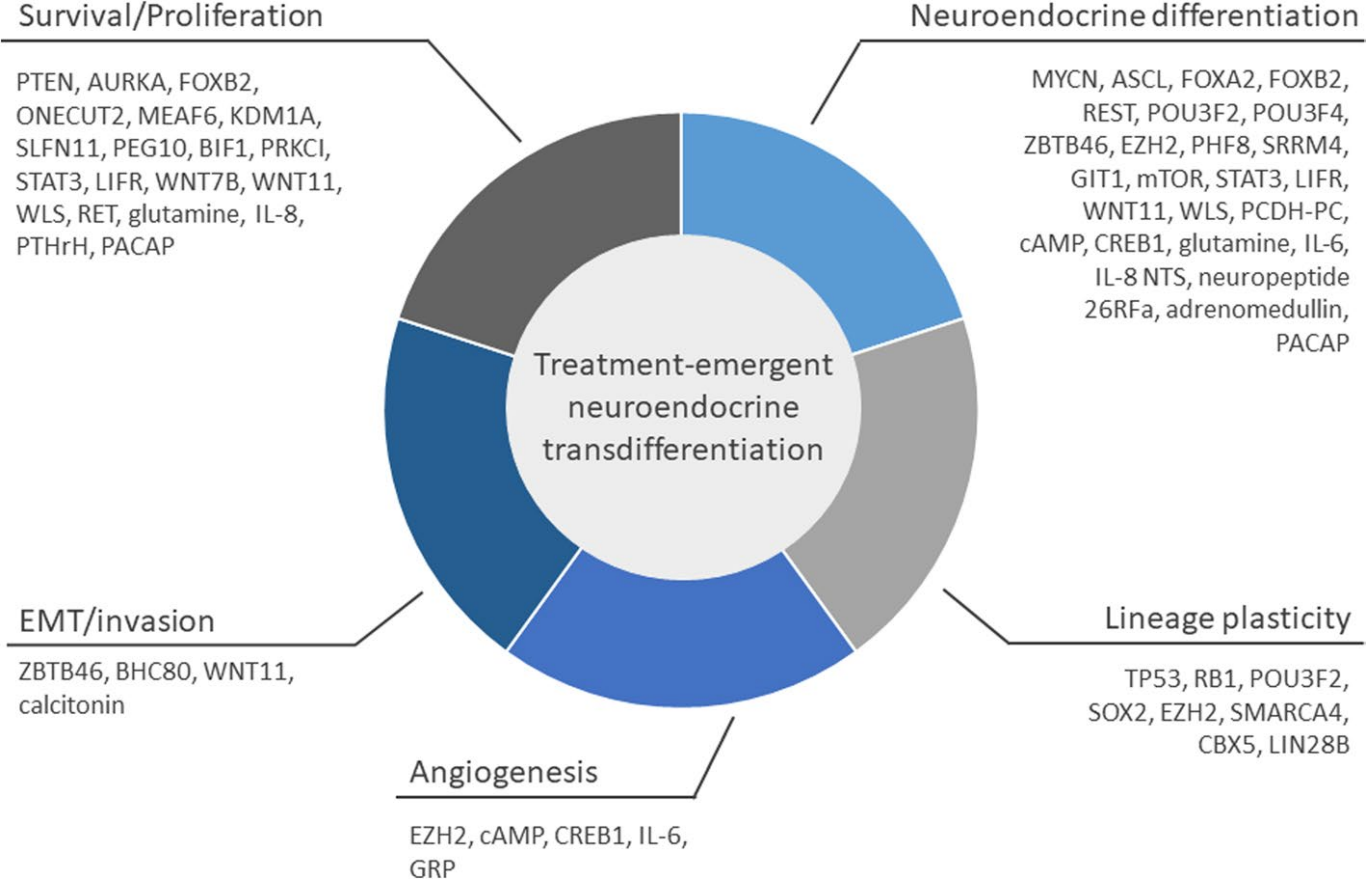
Key mechanisms contributing to t-NEPC transdifferentiation. Genes and proteins discussed in this review are ordered based on the effects of their respective aberrations in t-NEPC

The transdifferentiation from mCRPC to t-NEPC seems to be driven by epigenetic changes rather than genomic aberrations, though some point mutations and copy number aberrations might be indicative of AVPC. Importantly, NE differentiation alone is not sufficient for the development of this rapidly growing cancer: In fact, neuroendocrine cells in healthy prostate tissue are not characterized by enhanced proliferation and the induction of a neuroendocrine phenotype has been accompanied by reduced cell growth and proliferation in cell culture experiments [23, 24]. Therefore, changes in neuroendocrine markers are commonly accompanied by the deregulation of cellular signaling pathways involved in lineage-plasticity, stem-like behavior and epithelial-to-mesenchymal transition (EMT). This close connection of NED and plasticity has been illustrated by overexpression of the basal marker TROP2 and the EMT-inducer SNAIL in PCa cell models. Depending on genetic background, both have been shown to be sufficient to induce an NE phenotype individually [25, 26], indicating that NED, basal-like gene expression and EMT are interwoven in PCa. Moreover, the deregulation of epigenetic factors such as chromatin modulators and histone modification writers and readers is necessary for the reprogramming of the cancer cell’s phenotype [8]. Figure 3 depicts the key mechanisms contributing to t-NEPC development. A comprehensive overview of all genes and proteins presented in this paper is given in Table 1.

**Table 1:** Summary of factors involved in t-NEPC development

Gene (Protein, if not identical)	Aberration in t-NEPC	Effects	Experimental Setting/ Source	Reference
**Genetic aberrations**				
PTEN	Deletion	Survival and cell cycle progression	Patient tissue biopsies	[27, 28]
TP53	Mutation	Lineage plasticity	Patient tissue biopsies	[28]
RB1	Deletion		Patient tissue biopsies	[28]
AURKA	Amplification	N-MYC stabilization, cell cycle progression	Patient tissue biopsies	[29]
MYCN	Amplification	Attenuation of AR signaling, NE differentiation, DDR activation	Patient tissue biopsies	[29–31]
**Transcription factors**				
ASCL1	Induced expression	NE/pro-neural differentiation	Patient tissue biopsies	[32]
FOXA1	Reduced expression	Loss of Epithelial differentiation	Patient tissue biopsies	[33]
FOXA2	Upregulated expression	Pioneering transcription factor, promotion of NE differentiation	Patient tissue biopsies	[34]
FOXB2	Upregulated expression	Activation of WNT-signaling, NE marker expression	Patient tissue biopsies	[35]
NKX2-1 (TTF-1)	Upregulated expression	unknown	Patient tissue biopsies	[36]
NKX3-1	Reduced expression	Loss of Epithelial differentiation	Patient tissue biopsies	[37]
REST	Reduced expression, alternative splicing	De-repression of neuronal genes, NE differentiation	Patient tissue biopsies	[32]
ONECUT2	Upregulated expression	Attenuation of AR signaling, survival	Patient tissue biopsies	[38]
POU3F2 (BRN2)	Upregulated expression	NE differentiation, lineage plasticity	Patient tissue biopsies	[39]
POU3F4 (BRN4)	Upregulated expression	NE differentiation	Patient tissue biopsies, PDX	[40]
SOX2	Upregulated expression	Lineage plasticity	Patient tissue biopsies	[32]
ZBTB46	Upregulated expression	NE differentiation, EMT, inflammatory signaling	Patient tissue biopsies	[41]
**Epigenetic factors**				
EZH2	Upregulated expression	Angiogenesis, NE differentiation	Patient tissue biopsies	[19]
PHF8	Upregulated expression	NE differentiation	Patient tissue biopsies, PDX	[42]
KDM1A (LSD1)	Alternative splicing	Cancer progression, therapy resistance, immune signaling	Patient tissue biopsies, PDX	[43]
MEAF6	Alternative splicing	Proliferation, anchorage-independent cell growth	Patient tissue biopsies, PDX	[44]
SMARCA4	Upregulated expression	Cell cycle progression, aggressiveness	Patient tissue biopsies	[45]
CBX5 (HP-1a)	Upregulated expression	Repression of luminal differentiation	Patient tissue biopsies, PDX	[46]
**DNA repair pathways**				
PARP1	Upregulated expression	DDR	Patient tissue biopsies, PDX	[30]
SLFN11	Reduced expression	Cell cycle progression	Patient tissue biopsies	[47]
**Other nuclear factors**				
CCND1	Upregulated expression	survival	Cell model	[48]
	Reduced expression	unknown	Patient tissue biopsies	[49]
LIN28B	Upregulated expression	Stemness and pluripotency/lineage plasticity	Patient tissue biopsies	[21]
PEG10	Upregulated expression	Cell cycle progression, EMT	Patient tissue biopsies	[32]
SRRM4	Upregulated expression	NE differentiation	Patient tissue biopsies	[50]
GIT1	Alternative splicing, GIT1-A upregulation	Neural differentiation, cell adhesion	Patient tissue biopsies, PDX	[51]
BIF1	Alternative splicing, BIF1b/c upregulation	Survival	Patient tissue biopsies, PDX	[52]
BHC80	Alternative splicing, BHC80-2 upregulation	Cell growth and invasion	Patient tissue biopsies, PDX	[53]
**Signaling pathways**				
mTOR	Increased activity	NE differentiation, reduced growth, increased one-carbon pathway	NEPC cell model	[54]
PRKCI	Reduced expression	Cell proliferation, increased one-carbon pathway	Patient tissue biopsies	[55]
STAT3	Increased activity	NE differentiation, proliferation	Cell model	[23]
LIFR	Upregulated expression	NE differentiation, cell proliferation	Patient tissue biopsies	[56]
WNT7B	Upregulated expression	Tumor growth, stemness, drug resistance	Patient tissue biopsies	[35]
WNT11	Upregulated expression	Promotion of NE differentiation, survival migration	AR- independent Cell model and xenografts	[57, 58]
WLS	Upregulated expression	Promotion of Wnt signaling, proliferation, NE differentiation	Patient tissue biopsies	[59]
PCDH-PC	Upregulated expression	Wnt activation, NE differentiation	Patient tissue biopsies	[60, 61]
CREB1	Increased activation	NE differentiation, angiogenesis, epigenetic reprogramming	Cell model	[62]
RET	Upregulated expression	Tumor growth	Patient tissue biopsies	[63]
**Tumor microenvironment**				
cAMP	Increased concentration	NE differentiation, angiogenesis, epigenetic reprogramming	Cell model	[64]
Glutamine	Increased concentration	ATP production, proliferation, NE differentiation	Cell model	[65]
IL-6	Increased concentration	NE differentiation, angiogenesis	Cell model	[64]
IL-8	Upregulated expression	Survival, proliferation	Cell model	[66]
GRPR/bombesin	Upregulated expression	Angiogenesis	Cell model	[67]
BIRC5 (Survivin)	Upregulated expression	Survival	Patient tissue biopsies	[68]
NTS (Neurotensin)	Upregulated expression	NE differentiation	Cell model and xenograft	[69]
PTHrH	Upregulated expression	Cell proliferation, treatment-resistance	Cell model	[70]
CALCA (Calcitonin)	Upregulated expression	Invasion	Cell model	[71]
QRFP (Neuropeptide 26RFa)	Upregulated expression	NE differentiation, migration	Patient tissue biopsies	[72]
ADM (Adrenomedullin)	Upregulated expression	NE differentiation	Cell model	[73]
ADCYAP1 (Pituitary adenylate cyclase activating polypeptide)	Upregulated expression	Cell proliferation, NE differentiation	Cell model	[74]
GABA	Upregulated expression	GRP release	Cell model	[75]

For genes and proteins presented in this review key information regarding their aberration in t-NEPC and the cellular effects of these aberrations are summed up. The experimental setting refers to the most reliable type of data in which the respective aberration has been found (Patient tissue biopsies > PDX > xenografts > cell model)

### Genomic aberrations

Although the differences between t-NEPC and mCRPC seem to be more pronounced on the epigenetic and transcriptional level, there are characteristic genomic aberrations that have been found to be co-occurring or more frequent in t-NEPC and AVPC, in general [8, 19].

Genomic aberrations, especially those leading to the loss-of-function, of the tumor suppressor phosphatase and tensin homolog (*PTEN*) are amidst the most frequent findings in PCa. *PTEN* loss is continually found in localized disease and confers activation of the PI3 kinase/AKT pathway [27]. In metastatic disease, the abundance of aberrations in *PTEN* is significantly increased, especially in combination with deleterious variants in the well-known tumor suppressor genes *RB1* and *TP53* [27]. Although combined loss-of-function aberrations in*TP53, RB1* and *PTEN* are found in mCRPC, the combination of genomic aberrations in at least two of these genes is indicative of AVPC [27, 28]. Studies with combinatorial knock-outs (KO) in mouse models and PCa cell lines have confirmed that single KOs are not sufficient to introduce an NE phenotype. In contrast, the combination but not the single KOs of *TP53* and *RB1* has induced the growth of an AR-low NE-like tumor in both models [76, 77]. A triple KO model with *PTEN* loss has exhibited an even more aggressive growth with multiple metastases [76]. Although aberrations of these three genes do not seem to be directly involved in the induction of NE genes, they mediated increased lineage plasticity by upregulating SRY-box transcription factor 2 (SOX2) and enhancer of zeste homolog 2 (EZH2) (see below) [77]. As complexes of RB1 and transcription factor E2F directly repress SOX2 and EZH2, RB1 loss derepresses these genes thereby enabling reprogramming towards a stem cell-like state [76]. PTEN acts by suppressing the activity of the PI3K/AKT signaling pathway as well as by inducing G_1_ arrest, thereby inhibiting cell cycle progression. PTEN loss also contributes to genomic instability, for example by increasing replication stress [78]. In addition to the attenuation of apoptosis, loss of TP53 function has been shown to increase tumor vascularization [79].

Other genomic aberrations frequently found in t-NEPC include the amplification of aurora kinase A (AURKA) and N-MYC (encoded by *MYCN*) [29]. AURKA is a mitotic kinase regulating various mitotic events and thereby mitotic exit and cell cycle progression. Therefore, its amplification in cancer is associated with tumorigenesis and deregulated proliferation [80]. The transcription factor N-MYC is crucial in embryonic development and for maturation of the central nervous system [81]. Its deregulation is primarily associated with tumors of the central nervous system, but N-MYC has also been found to be highly enriched in t-NEPC tumors (40% vs. 5% in adeno- PCa) [82]. By direct binding to their promoters, N-MYC regulates the transcription of DNA damage response (DDR) pathway-associated genes, including PARP1/2, BRCA1, RMI2 and TOPBP1 [30]. Notably, increased expression and activity of DDR factors has been associated with resistance to chemotherapy before [83, 84]. Thus, N-MYC driven aberrant regulation might actually influence treatment response in t-NEPC as well. In addition, N-MYC has been found to attenuate AR signaling and directly activate EZH2, thereby contributing to epigenetic reprogramming [31]. Interestingly, AURKA directly binds to N-MYC, thereby increasing its stability via inhibiting the interaction with the E3 ubiquitin ligase FBXW7, a mechanism that has been examined in detail in neuroblastoma [29, 85].

Apart from gene amplification, AURKA and N-MYC expression can also be increased by reduced protein degradation mediated by *TP53* mutation and microRNA-25 [86]. Moreover, the AR has also been described to bind to the *AURKA* gene and increase its expression in CRPC with AR amplification [87].

### Transcription factors

Achaete-Scute Family BHLH Transcription Factor 1 (ASCL1) is a pioneer transcription factor binding to closed chromatin regions and is directly involved in neuronal lineage differentiation [88]. ASCL1 has been shown to be induced upon androgen deprivation in LNCaP cells with an accumulation in the nucleus [20]. In t-NEPC patient cohorts, ASCL1 expression has been upregulated in comparison to mCRPC [32]. ASCL1 expression is generally associated with neuronal differentiation and upregulation of NE markers [20]. Small cell prostate cancer and small cell lung cancer exhibit transcriptional similarities including ASCL1 expression [89]. In small cell lung cancer, ASCL1 has been identified as a downstream factor of BRN2 and has been implicated in the induction of RET kinase, however these pathways have not been proven in t-NEPC, yet [90, 91]. In t-NEPC, ASCL1 has indirectly induced the expression of the cell adhesion protein CEACAM5 [92]. Importantly, ASCL1 nuclear localization has persisted in PCa cell culture models, even after reversal of NE differentiation by androgen supplementation. Therefore, ASCL1 has been suggested to mediate a hybrid state, in which NE and epithelial markers are co-expressed, when cells have been exposed to intermittent androgen deprivation [20].

Pioneering lineage-defining transcription factor FOXA2 has been described as a marker for t-NEPC with similar specificity to NE markers CHGA and SYP, but with enhanced sensitivity [34]. Upregulation of FOXA2 has been found to be at least partially mediated by increased activity of PHF8, which removes repressive histone marks from the *FOXA2* promoter [42]. In the transgenic mouse model, FOXA2 has been involved in enhancing the expression of a subset of HIF-1α target genes by direct interaction with the transcription factor under hypoxia and in contributing to NE differentiation [93].

FOX transcription factors primarily regulate cellular plasticity, and are suggested to be a key factor in epithelial to NE differentiation, including FOXA1 and FOXB2 [33, 35]. The critical role of FOXA1 in PCa is the regulation of the AR pathway, but its role in t-NEPC remains controversial [33, 94]. Kim et al. observed a loss of FOXA1 in t-NEPC compared to mCRPC datasets and identified subsequent induction of IL-8 as a mechanism of increased expression of the NE marker ENO2 [33]. However, a recent study by Baca et al. found a maintained FOXA1 expression in t-NEPC, while the FOXA1 cistrome was reprogrammed [94]. Next, the transcription factor FOXB2, which is associated with neuronal development, has been found to be upregulated in advanced PCa including t-NEPC. FOXB2 has been involved in the emergence of t-NEPC due to its role in activating WNT-signaling independently of β-catenin, for instance by activation of WNT7B. In addition, overexpression of FOXB2 has been sufficient to induce NE marker expression and a neuron-like morphology in LNCaP cells [35]. Another layer of growing complexity is brought by recent studies that identified several non-coding RNAs, such as microRNA-194 and LINC00261, that promote neuroendocrine transdifferentiation by regulating expression of FOXA1 and FOXA2, respectively [95, 96].

The transcription factor NKX3-1 is activated by AR signaling and indicates prostate epithelial differentiation. Its expression is decreased in AR-independent PCa and t-NEPC [11]. *NKX2-1*, in contrast, encodes the thyroid transcription factor-1 (TTF1) protein, which has formerly been considered a marker of lung cancer. However, TTF1 has also been shown to be expressed in about 50% of a cohort of t-NEPC patients [36]. Although it has been associated with enhanced proliferation and shorter overall survival (OS) [36], the function of TTF1 in t-NEPC has not been unraveled, yet. Findings in other NE tumors indicate that TTF1 might generally contribute to NE reprogramming [89].

RE1 silencing transcription factor (REST), a transcriptional repressor restricting the expression of neuronal genes, is expressed in prostate epithelial cells. Different pathways have been identified that lead to a repression of REST in t-NEPC. While AR signaling indirectly enhances REST stability by inhibiting the ubiquitin ligase subunit BTRC and thus prevents degradation of REST, inhibition of AR signaling destabilizes the REST protein [97]. In addition, the splicing factor Serine/Arginine Repetitive Matrix 4 (SRRM4), which is associated with neuronal differentiation, converts the active *REST* transcript to its inactive isoform REST4, which is no longer capable of repressing its target genes [98]. Lastly, cell stress such as hypoxia or inhibition of AKT signaling have also been shown to inhibit REST [99]. As a consequence of lost REST expression, neural genes such as *CHGA* and *SYP* as well as *AURKA* are increasingly expressed and in turn promote the NE-phenotype [97]. Of note, knock-down of *REST* in the castration-resistant C4-2B cell model has been shown to result in a G_1_ arrest, which can been rescued by *TP53* inactivation [100].

Another transcription factor that is repressed by REST is ONECUT2, which has recently been identified to be involved in the progression to AR-independent disease. Interestingly, ONECUT2 increases the expression of PEG10 and inhibits the expression of AR and its downstream transcription factor FOXA1, thereby contributing to a NE phenotype [101]. As a mechanism of action of ONECUT2, the induction of hypoxia signaling by activation of HIF-1α via SMAD3 has been proposed to drive NED in the PCa cell model [38].

A family of transcription factors that is essential in neurogenesis are Class III (Pit-Oct-Unc)-domain/Oct proteins (POU3F) [102]. *POU3F2* and *POU3F4* (also known as BRN2 and BRN4) have both been found to be upregulated in t-NEPC [39, 40]. Cell models suggest that the combinatorial KO of *TP53* and *RB1*, AR inhibition as well as overexpression of N-MYC can cause the upregulation of BRN2 and BRN4 [39, 77]. Additionally, BRN2 overexpression upregulates BRN4 [40]. BRN4 overexpression induces SOX2 and, in turn, SOX2 sites have been identified in the BRN4 promotor. This suggests that a regulatory feedback loop is activated in t-NEPC. Due to the direct interactions of BRN2 and BRN4, it seems likely that both are involved in SOX2 regulation [40]. Overexpression of BRN2 has been sufficient to induce the expression of NE markers such as SYP and NCAM1 in a prostate cancer cell model [39]. Together with SOX2, BRN2 has been shown to activate further genes in stem and progenitor cells that are not directly linked to NE differentiation, but more generally to tumorigenesis and lineage plasticity [103].

SOX2 is a central factor in embryonal development and maintenance of pluripotency [104]. In healthy tissue or low-grade adenocarcinoma, AR represses SOX2 and maintains the differentiated state. However, SOX2 expression can be induced by direct binding of BRN2 and E2F, among others, to the SOX2 promoter [39, 105]. The activity of E2F is increased in cells with combinatorial *TP53* and *RB1* loss of function. Thus, loss of these tumor suppressors has been associated with increased SOX2 expression and lineage plasticity as mentioned before [77]. SOX2 is considered to promote a plastic cell state that facilitates the acquisition of further phenotypical changes such as expression of the NE marker SYP, but is not considered to be sufficient to confer enzalutamide resistance on its own [106].

SAM Pointed Domain Containing ETS Transcription Factor (SPDEF) is a transcription factor of the ETS family specific for prostate epithelial cells. It has been found to be a transactivator of PSA and is generally associated with regulation of AR activity. In accordance, experimental data have shown a decrease in SPDEF expression in t-NEPC patient samples and cell lines [41]. DNA hypermethylation has been identified as an important mechanism in SPDEF silencing [19]. SPDEF has been found to bind to the promoter of the transcription factor zinc finger and BTB domain containing 46 (ZBTB46) which is upregulated in t-NEPC. ZBTB46 promotes NED by enhanced expression of leukemia inhibitory factor (LIF) and nerve growth factor in vitro [107, 108].

### Deregulation of epigenetic factors

**Fig. 4:**
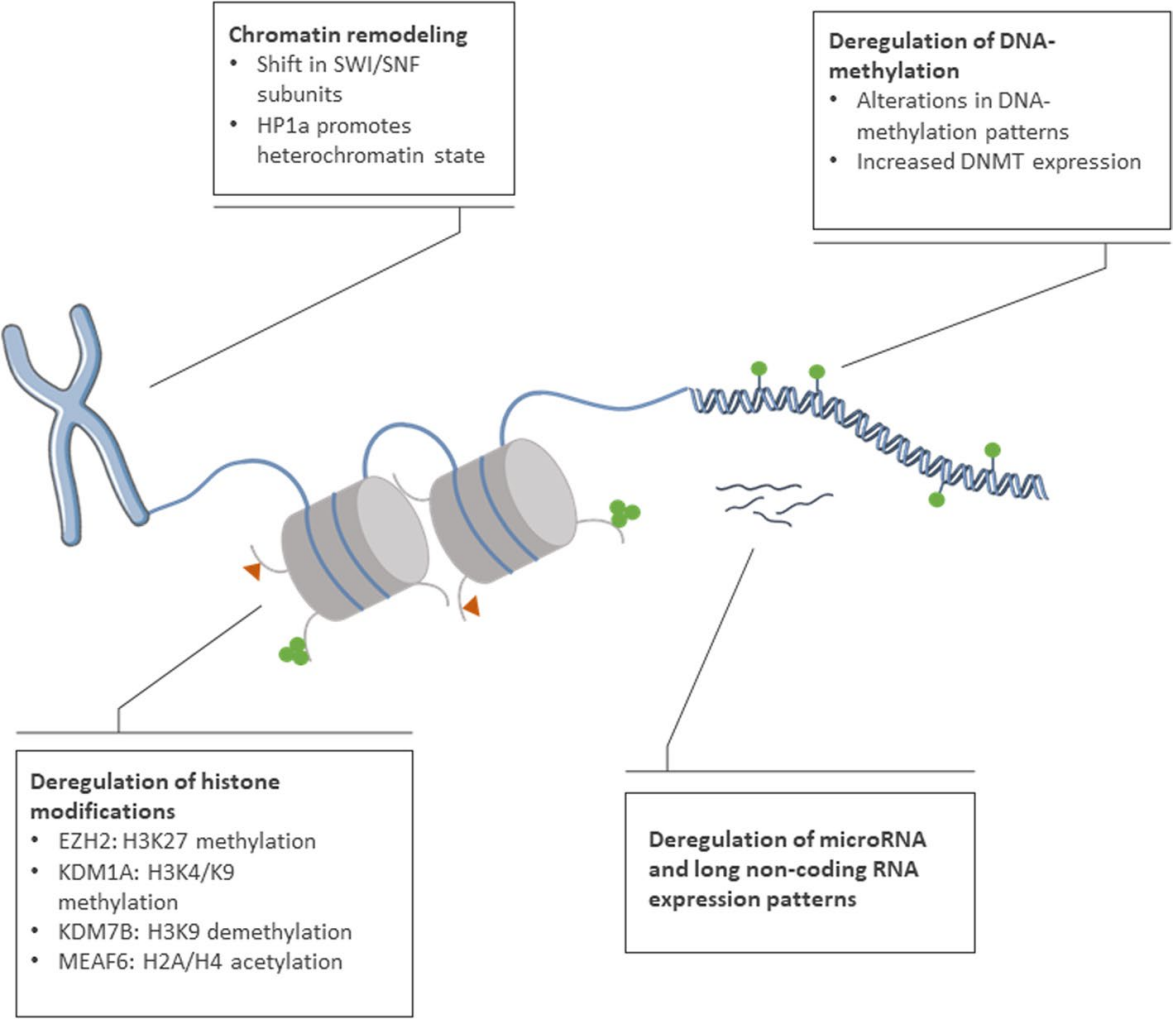
Overview of epigenetic alterations in t-NEPC. Factors contributing to epigenetic deregulation are assigned to their respective mechanism of action; methylation of histone tails or DNA is indicated by green dots, histone acetylation is represented by orange triangles

Epigenetic regulation of gene expression includes the methylation of DNA, histone modifications and non-coding RNA-species. An overview of epigenetic alterations in t-NEPC is given in Fig. 4. Generally, malignant disease is characterized by global hypomethylation of the DNA compared to the benign tissue and hypermethylation of specific tumor suppressor loci [109]. This is also observed in PCa. Recently published whole genome bisulfite sequencing data have indicated that mCRPC and t-NEPC can be distinguished based on DNA methylation patterns, which emphasize the relevance of epigenetic mechanisms in t-NEPC development [110, 111]. DNA methylation is mainly governed by the activity of DNA methyltransferases (DNMT) and TET enzymes, which remove methyl groups. In prostate cancer, expression levels of the three DNMTs have been found to continually increase with disease progression [112]. However, so far no distinct t-NEPC expression pattern has been identified.

Epigenetic regulation is also conferred by the posttranslational modification of histones. These individual methylation modifications on histones can have different roles (activating or silencing) depending on the genetic context, thereby altering the chromatin structure and influence the accessibility for transcription factors [113, 114]. EZH2 is a histone-lysine N-methyltransferase that catalyzes the methylation of lysine 27 in histone 3 (H3K27). This mark represses the transcription of affected genes [115]. Expression of EZH2 has been shown to be upregulated in t-NEPC, directly mediated by an increased activity of E2F transcription factors [116]. PKA/CREB activation has also been found to cause elevated EZH2 levels in a t-NEPC cell model [62]. EZH2 targets comprise genes with diverse functions, but EZH2 activity is generally associated with cell fate determination. Concretely, increased EZH2 expression has been shown to promote NE marker expression. Additionally, EZH2 directly contributes to tumor angiogenesis by reducing the levels of the angiogenesis inhibitor TSP1 [62].

Histone demethylase KDM7B, also known as PHF8, binds to di- and tri-methylated histone H3 lysine 4 (H3K4me2/3), associated with transcriptional activation, and removes the repressing marks H3K9me1/2 and H3K27me2. Thereby, PHF8 promotes an active chromatin state that allows transcription of the respective genes [117]. Hypoxia and c-Myc signaling both increase the expression of PHF8 in PCa [118]. In CRPC, PHF8 has been shown to act as a coactivator of AR [119]. Thus, the overexpression of PHF8 has been shown to cause resistance of LNCaP cells to enzalutamide [42]. Importantly, overexpression of PHF8 has also been shown to enhance the expression of NE markers such as NSE in a mouse model. Mechanistically, PHF8 removes repressive marks from the FOXA2 promoter, which causes induction of FOXA2 expression. Co-expression of both, PHF8 and FOXA2, has been demonstrated to be much higher in t-NEPC patient-derived xenografts (PDX) and patient samples compared to adenocarcinoma [42].

Another histone demethylase, KDM1A, also known as LSD1, removes the activating marks H3K4me2/1. Thereby, LSD1 promotes a repressive chromatin state and regulates gene expression in stem cells [120]. In CRPC, LSD1 has been found to be upregulated and promote AR-independent survival. Remarkably, LSD1 exhibits demethylase-independent functions in transcriptional regulation. In cell models, LSD1 has mediated expression of several genes mainly involved in mitosis and replication [121]. In addition, LSD1 has been shown to be a target of the splicing factor SRRM4 in PCa. The LSD1 + 8a splice variant, resulting in inclusion of a novel 12 nucleotide micro-exon (exon 8a) and alternative substrate specificity and regulation to canonical LSD1 isoform, has been found exclusively in t-NEPC samples where it upregulates the expression of genes associated with cancer progression and therapy resistance [43, 122].

The MYST/Esa1-associated factor 6 (MEAF6) is a subunit of the NuA4 histone acetyltransferase complex, which mediates a transcriptional active chromatin state by acetylation of H4 and H2A [123] In t-NEPC, expression of the neural-specific isoform MEAF6-1 has been described to be increased due to the enhanced expression of the splicing factor SRRM4 [44]. MEAF6-1 has been shown to promote anchorage-independent cell growth and proliferation in t-NEPC cell models and xenografts, possibly through upregulating *ID1* and *ID3* genes, which inhibit differentiation and promote proliferation [124]. However, neither MEAF6-1 nor MEAF6-2 have been capable of inducing NE differentiation in this cell model, indicating that MEAF6 is not a driver of NE-differentiation. The increase in the MEAF6-1 splice variant has been suggested to be rather a facilitator of cell proliferation [44].

Apart from histone modifications, the conformation of the chromatin can be rebuild by ATP-dependent mammalian SWI/SNF chromatin remodeling complexes, also known as Brg/Brahma-associated factor (BAF) complexes [125]. In fact, the expression of the SWI/SNF subunits BAF53B and BAF45B, which are considered to be neuron-specific, has been observed in t-NEPC patient samples. The mechanism mediating the expression of BAF53B/45B has not been identified, yet, but loss of REST expression has been excluded. Knock-down of neither BAF53B nor BAF45B has shown an impact on cell proliferation of organoids modelling NEPC. Therefore, these subunits may rather be passengers of t-NEPC development than drivers. SMARCA4 is another SWI/SNF subunit upregulated in t-NEPC. Its expression is correlated with worse OS and more aggressive disease. At least in part, this has been led back to its involvement in lineage plasticity [45].

The heterochromatin protein 1α (HP1a, encoded by *CBX5*), is involved in the silencing of target genes by promotion of the heterochromatin state [126]. In a PDX model of transdifferentiation from prostate adenocarcinoma to t-NEPC, *HP1a* has been identified as one of the earliest upregulated genes after castration. HP1a has further continued to increase during t-NEPC development. Increased expression of HP1a has also been confirmed in published cohorts of t-NEPC patients. Although overexpression of HP1a alone was not sufficient to induce NED in castration-resistant LNCaP, expression of the NE markers NSE and NCAM1 was significantly higher after enzalutamide treatment of HP1a-overexpressing versus control cells. Chromatin immunoprecipitation results suggest that HP1a acts by mediating increased repressive H3K9 histone methylation at *AR* and *REST* promoter regions. Accordingly, AR and REST mRNA and protein levels have been found to be decreased in HP1a-overexpressing cells [46].

Altered expression of non-coding RNA species, including microRNAs and long non-coding RNAs, shape the epigenetic landscape of t-NEPC. For instance, RNA sequencing of patient samples and PDX has revealed significant changes in microRNA expression patterns in t-NEPC compared to adenocarcinoma. Interestingly, microRNAs targeting *MYCN*, *AURKA, STAT3, E2F1* and *EZH2*, among others, have been found to be down-regulated while microRNAs targeting *RB1* are increased [127]. Additionally, long non-coding RNAs modulate gene expression by various mechanisms and exhibit specific expression signatures in t-NEPC. Thus, long non-coding RNAs have been found to contribute to enhanced expression of SOX2 and the loss of TP53 [128].

### Deregulation of DNA repair pathways

The role of DDR defects for the development of t-NEPC has not been conclusively clarified, yet. Beltran et al. have not detected significant differences in the frequency of DDR gene mutations in t-NEPC compared to mCRPC patients [110]. In contrast, Aggarwal et al. have reported a reduced frequency of DDR mutations in t-NEPC which they have described as close to “mutually exclusive” with t-NEPC [11]. Thus, further data from larger cohorts is urgently needed to clarify the relevance of DDR mutations in t-NEPC.

Recently PARP-inhibitors have been approved for therapy in PCa patients with DDR pathway mutations and have already been used to treat t-NEPC patients with such aberrations, though only with limited success [129]. PARP1 is a chromatin associated enzyme modifying various nuclear proteins by poly-ADP-ribosylation, but it also functions as transcriptional coactivator for E2F1 [130]. In t-NEPC cell models and xenografts, PARP inhibitors have been shown to decrease cell proliferation and tumor growth. PARP inhibitors have also suppressed the expression of NE markers, at least partially by inferring with N-MYC and E2F activity [131]. As a coactivator of N-MYC, PARP1/2 may regulate DDR genes in t-NEPC [30]. In addition, overexpression of the stem-cell marker TROP2 increases PARP1 expression and induces expression of NE markers in vitro. Of note, PARP inhibition has led to a significant decrease of tumor growth and NE marker expression in the same setting [25]. Moreover, combined loss of PTEN and TP53 has been shown to sensitize cells to PARP inhibitors [132]. However, expression of the DDR-related gene *SLFN11,* which has been associated with enhanced sensitivity to PARP inhibition, is less-frequent in patient samples of t-NEPC compared to mCRPC [47].

### Deregulation of other nuclear factors

Cyclin D1 (CCND1) functions in a complex with cell cycle-dependent kinases 4/6 to promote cell cycle progression. The role of CCND1 in t-NEPC remains elusive, as contrary results on its expression have been found. Exploration of mRNA and microRNA expression levels in a cell model of NED has revealed upregulation of CCND1, while no significant changes have been detected in other cyclins. This altered expression level has been caused by decreased expression of the microRNA-17 family, which bind to the *CCND1* mRNA [48]. In contrast, Tsai et al. reported the loss of CCND1 expression to be indicative of small cell-like PCa in patient tissue, as 88% of samples classified as small cell carcinoma have been observed to be CCND1-negative compared to less than 10% of the adenocarcinomas [49]. Additionally, a recent study in de novo NEPC identified loss of CCND1 expression in patient tissue [133]. Possibly, CCND1 loss is predominantly involved in de novo-emergence of NEPC. Despite its function in cell cycle progression, CCND1 also has kinase-independent functions, as it has been shown to act as a co-repressor of AR signaling in PCa [134]. Together with CDKN2A, CCND1 expression levels have also been suggested as a biomarker for RB1 functional status [49].

The nuclear protein LIN28B is known for its ability to inhibit the maturation of pri-let7 transcripts, thereby maintaining the pluripotent state of stem cells. When expressed in cancer cells, LIN28B promotes the expression of genes associated with embryogenesis and lineage plasticity [135]. Recently, Lovnicki et al. reported the expression of LIN28B in a subset of t-NEPC patients. Evaluation of LIN28B in the studied cell model suggested a pathway in which the inhibition of let-7 transcripts by LIN28B enables the expression of High Mobility Group AT-Hook 2 (HMGA2), a transcriptional activator of the SOX2 gene. A positive correlation of LIN28B and SOX2 expression has been confirmed in patient datasets. As LIN28B was only expressed in about half of the t-NEPC samples, the authors suggested that this might indicate the development of t-NEPC by two different pathways. LIN28B-high cells might evolve from an intermediate stem-like state that undergoes NE-differentiation, whereas the LIN28B-low cells might be a result of direct transdifferentiation. However, they also speculated that LIN28B expression might be lost after NE-differentiation, meaning that the two groups of LIN28B high and low t-NEPC represent different stages of the transdifferentiation process [21]. Sequencing results from mouse KO models under NHA indicate that LIN28B expression is not enabled by *PTEN* KO alone, but by *TP53* double KO or *TP53* and *RB1* triple KO [76].

The retrotransposon-derived protein PEG10 is a paternally expressed imprinted gene present in adult as well as embryonic tissues. Expression of PEG10 has been repressed by the AR which is supported by the observation that PEG10 is de-repressed in response to NHA treatment [136, 137]. Additionally, E2F transcription factors have been identified as direct regulators of PEG10 and their activity is increased in t-NEPC, following TP53 and RB1 loss of function [76]. In t-NEPC, PEG10 is thought to be involved in cell cycle progression following TP53 loss as well as in motility and EMT as it has been found to promote TGF-β signaling [136].

SRRM4 is a splice factor associated with neuronal development that has been found to be upregulated in t-NEPC [50, 138]. Genes that have been shown to be alternatively spliced by SRRM4 in t-NEPC include GIT1, REST, BIF-1, BHC80, LSD1, and MEAF6 [43, 44, 51–53, 97]. The upregulation of the GIT1-A splice variant and downregulation of the GIT1-C variant have been identified in t-NEPC patient samples, PDX and cell lines and has been associated with morphogenesis, neural function and epigenetic regulation [51]. Next, when spliced by SRRM4, the transcriptional repressor REST has been shown to be converted to its inactive isoform REST4, causing a loss of its repressive effect on neuronal genes [97]. By alternative splicing of the *BIF-1* gene, SRRM4 has enhanced the expression of anti-apoptotic BIF-1b and BIF-1c variants, thereby contributing to cell survival during AR pathway inhibition [52]. Proliferation of t-NEPC has also been reported to be increased by the BHC80-2 splice variant which, in contrast to the BHC80-1, has histone demethylase-independent functions and indirectly stabilizes tumor-promoting cytokines [53].

### Deregulation of signaling pathways

#### mTOR signaling pathway

The PI3K/AKT pathway is one of the main regulators of cell survival, proliferation and metabolism. The pathway is deregulated in several solid cancer entities and mostly due to common *PTEN* loss, activated PI3K/AKT signaling is already present in hormone-sensitive PCa [139]. The mammalian target of rapamycin (mTOR) is a downstream factor of AKT signaling and a key regulator of metabolism and biosynthesis, which might be of increased importance in t-NEPC. However, the exact consequences of its activation for NED are not completely understood. Expression of protein kinase C iota (PRKCI) has been found to be reduced in t-NEPC compared to mCRPC in patient tissue samples. In vitro, loss of PRKCI contributes to the activation of mTORC1 via loss of phosphorylation of the mTORC1 regulator LAMTOR2 [55]. A recent study by Kanayama et al. suggested that the expression of constitutively active mTOR was sufficient for the induction of a NE-morphological changes and NSE expression in LNCaP cells. However, the expression was also found to induce a growth arrest, mediated at least partially by the mTOR targets IRF1 and CDK inhibitor p21. Of note, IRF1 was not involved in the induction of NSE [54].

In addition, increased activity of the transcription factor ATF4 causes an increase in the serine, glycine, one-carbon pathway, downstream of mTOR activation. This leads to elevated levels of cell metabolites, including the methyl-donor S-adenosyl methionine, and ultimately to increased DNA methylation, for example of AR target genes. This emphasizes the role of mTOR not only in cell metabolism, but also as a potential facilitator of epigenetic reprogramming. ATF4 seems to be also involved in the expression of NE and basal markers, but a distinct mechanism has not been identified yet [55].

Another target of mTOR is the transcription activator STAT3, which has also been associated with NE-differentiation [54]. For instance, STAT3 has been shown to be activated by IL-6 and LIF receptor (LIFR) in androgen-deprived PCa cell models [23]. Indeed, expression of LIFR is increased in t-NEPC as compared to adenocarcinoma and correlates with elevated NE marker expression in patients’ tissues. In vitro, androgen withdrawal has been shown to increase LIFR expression, while overexpression of LIFR enhances NE marker expression. By activation of STAT3, LIFR shows increased expression of succinate-CoA ligase GDP-forming beta subunit SUCLG2, which, in turn, enhances cell proliferation and facilitates nucleotide synthesis in LNCaP cells [56]. However, SUCLG2 expression has been found to be not significantly different between t-NEPC and high-grade adenocarcinoma patients [56], indicating that LIFR activation and STAT3 signaling are not exclusive regulators of SUCLG2 in t-NEPC. LIF, the ligand of LIFR, is increased in CRPC patients upon AR inhibition and may be part of a positive feedback loop activating the transcription factor ZBTB46, which has also been shown to be activated in t-NEPC [107].

#### WNT signaling pathway

The WNT signaling pathway is a major regulator of development and stemness in various solid tumors [140]. In PCa, WNT signaling is associated with therapy resistance and cancer stem cell renewal [141]. For instance, activation of the noncanonical WNT pathway has been identified in patients progressing on AR-inhibition compared with untreated patients [142]. Increased expression of FOXB2 upregulates the expression of WNT7B in t-NEPC. This enables the induction WNT pathway activity irrespective of β-catenin [35]. WNT11 is another WNT ligand that has been identified to be upregulated upon androgen depletion in vitro. WNT11 expression induces the expression of NE-markers and the transcription factor ASCL1, but these changes have only been observed in malignant and not in benign prostate cells. Thus, further aberrations seem to be necessary for this WNT11 activity [57]. Additionally, WNT signaling and NE-differentiation have been facilitated by the WNT carrier protein WLS. WLS is repressed by the AR in adenocarcinoma, but is increasingly expressed upon AR inhibition and t-NEPC transdifferentiation [59]. Another inducer of WNT signaling is protocadherin-PC (PCDH-PC), which has been reported to be specifically upregulated in castration-resistant disease. Of note, in a PCa cell model, PCDH-PC overexpression has been accompanied by increased WNT signaling and NE marker expression [60, 61].

#### cAMP signaling pathway

cAMP has been one of the firstly identified inducers of NED, promoting morphological changes of the cells as well as the production and secretion of NE-markers such as NSE, SYP and CHGA [143]. Increased cAMP levels may result from ADT, beta-adrenergic stress signaling or copy number gains in adenylate cyclase 8 [28, 62, 144]. As a consequence of elevated cAMP levels, the PKA-CREB1 pathway is activated. Targets of CREB1 include the pro-angiogenic key driver VEGF, EZH2, HDAC2 and the NE-marker ENO2 [62, 144].

#### RET signaling pathway

The receptor tyrosine kinase and protooncogene RET is associated with the development of the nervous system and has been upregulated and hyperphosphorylated in neuroendocrine malignancies including t-NEPC [145]. In patient samples, RET expression positively correlated with NE marker expression and negatively correlated with AR target gene expression. RET knock-down or inhibition in a t-NEPC cell model reduced the phosphorylation of ERK1/2, indicating an activation of MAP kinase pathway by RET. The inhibition of RET by small molecule inhibitors decreases tumor growth of t-NEPC cell lines, 3D cultures and xenografts [63].

### Signaling in the tumor microenvironment (TME)

#### Cancer-associated fibroblasts

Cancer-associated fibroblasts are fibroblasts in the tumor stroma that contribute to cancer cell growth and proliferation, for instance by cytokine secretion. In cell culture experiments, treatment with NHA has also been found to affect prostate fibroblasts. As a consequence of increased DNMT activity upon NHA stimulation, these cells downregulate the expression of the RAS inhibitor RASAL3 and increase the release of glutamine. RASAL3 promoter hypermethylation has also been demonstrated in patient samples. In the tumor cells, increased extracellular glutamine concentration enhances glutamine uptake, ATP production and cell proliferation. Additionally, increased glutamine leads to the activation of mTOR and NE markers such as ENO2 [65].

#### Cytokines

Pro-inflammatory cytokines in the TME can mediate NE differentiation. For example, tumor-associated macrophages release IL-6, when stimulated with bone morphogenic protein-6 which, in turn, is secreted by PCa cells [146]. In addition, increased IL-6 secretion has been found in cancer-associated fibroblasts from PCa biopsies [147]. IL-6 initiates NED by inducing expression of the NE markers CHGA and ENO2 through STAT3 and MAPK pathways [23, 64]. Additionally, IL-6 promotes angiogenesis by up-regulating VEGF via PI3K/Akt signaling [147]. Moreover, IL-8 is expressed in benign as well as malignant NE cells [148]. As FOXA1 directly represses IL-8, loss of FOXA1 expression in t-NEPC has been considered the cause of an increased expression of IL-8. Effects of paracrine and autocrine IL-8 stimulation include cell survival and proliferation as well as NED mediated by MAPK and STAT3 signaling [33, 66].

#### Neuroendocrine peptides

T-NEPC tumor cells themselves release different compounds that have been described to initiate or promote NED in neighboring non-NEPC cells. For instance, the NE peptides, some of which are broadly used as NE-markers, exhibit distinct autocrine and paracrine functions in the TME. Culture of androgen-resistant PCa cells with NEPC-conditioned medium has been shown to confer a survival advantage upon NHA treatment [149]. Several single neuropeptides have meanwhile been identified that are secreted by NE and t-NEPC cells and stimulate survival, proliferation, migration and angiogenesis. These include survivin, gastrin-releasing peptide (GRP, bombesin), neurotensin, parathyroid hormone-related protein, serotonin and calcitonin [68, 150]. In addition, different peptides such as neurotensin, neuropeptide 26RFa, adrenomedullin and pituitary adenylate cyclase activating polypeptide have induced NED in PCa cell models [69, 72–74]. Neurotensin, for instance has been found to be upregulated in castration-resistant xenografts inducing the expression of NE markers and ASCL1 via its receptors NTSR1 and NTSR3 [69]. In addition, also EGFR and IGF1-R have been found to be involved in NE peptide signaling by activating, for example, focal adhesion kinase, ERK and PI3K/Akt signaling [149, 150]. This is, however, not specific for NED. In addition to the expression of these NE-peptides, calcium-dependent secretion and the expression of T-type calcium channels have been upregulated in a NE-cell model, which is of major importance for autocrine and paracrine cell stimulation [151]. Moreover, AR-knockdown has caused an increase in the secretion of the neurotransmitter GABA and a slight upregulation in the GABBR1 receptor in vitro. Stimulation of the GABBR1 receptor, in turn, led to an increase in GRP secretion, which is associated with enhanced migration and angiogenesis [67, 75, 152].

#### Extracellular vesicles

Extracellular vesicles containing proteins and nucleic acid, among others, within a lipid membrane are released by cells and, for instance, serve cell-cell communication. Vesicles released from androgen-independent cancer cells have been shown to promote androgen-independence in androgen-dependent cells [153]. Moreover, extracellular vesicles released by t-NEPC or cell lines that resemble this subtype have been shown to contain, for instance, the transcription factors BRN2 and BRN4. Incubation of a non-NE cell line with these vesicles has altered the expression of NE markers and AR [40]. Caveolin-1 is another cargo of extracellular vesicles that has been identified to confer stemness and EMT in vitro, thereby promoting lineage plasticity [154].

## Conclusion and future directions

The emergence of NEPC is a major challenge in the nomenclature, diagnosis, classification and treatment of advanced PCa due to increased aggressiveness and a lack of effective treatment options. On the inter-patient as well as intra-patient level, t-NEPC presents as a heterogeneous disease. This includes the presence of histologically and molecularly mixed tumors as well as varying degrees of manifestation of different traits such as cellular stemness and plasticity. Recent investigations have brought first light into the growing complexity underlying the nature of t-NEPC development. As summarized in this review, different biological mechanisms and molecular determinants contributing to the manifestation of t-NEPC, have been identified and the mechanism of transdifferentiation from adenocarcinoma to t-NEPC is broadly accepted.

Mechanisms driving the development of t-NEPC are complex and analyses of patient samples have revealed genomic aberrations as well as significant epigenetic, transcriptional and posttranslational changes. Lineage plasticity, enhanced proliferation and EMT belong to the central features of t-NEPC.

Clinical observations as well as experimental studies point out that the deregulation of key factors involved in the control of cell cycle progression, such as TP53, RB1, PTEN, CCND1 and AURKA is invariably observed in t-NEPC tumors. Deregulation of these factors paves the way for unhindered accumulation of genomic aberrations in individual tumor cells [155]. Extensive deregulation of cell cycle control is a key event in the development of t-NEPC and seems to be one of the main sources of intra-tumoral heterogeneity and to contribute to therapy resistance found in t-NEPC.

Beyond this generic phenomenon of cancer progression, several studies show that the expression and activity of several proteins involved in transcriptional and epigenetic regulation are often altered in t-NEPC. Among others, this includes the induction of MYCN, ASCL1, FOXA2, SOX2, EZH2, PHF8 expression as well as down-regulation of FOXA1 or NKX3-1. It is well established that for untransformed, somatic cells the transition between different cell states during development is governed by transcription factor-induced reprogramming which also involves genome-wide changes to the chromatin [156]. We agree with the concept that this biological principle may also be true for transdifferentiation of NEPC, with the fundamental difference that the acquisition of a new cell state is the result of e.g. selection pressure of constant anti-androgen therapy [11, 157, 158]. This selection pressure will select for mutations, chromosomal rearrangements and epigenetic patterns that mediate the transition to a cell state which is independent of AR activity or otherwise helps to evade treatment-mediated cytotoxicity.

Today, several individual factors contributing to NE differentiation have been identified. However, the interplay of the different factors as well as the temporal order of genetic and epigenetic events causing the transdifferentiation still requires further clarification. Indeed, the heterogeneity of the disease may be caused by a varying influence of the described mechanisms of action. Therefore, a prioritization of distinct features and mechanisms such as stemness or epigenetic reprogramming is difficult at the current stage.

Continuation of research of t-NEPC is urgently needed, as a detailed knowledge of the processes underlying NED will support the development of reliable biomarkers and novel therapies for this lethal disease. Enhanced understanding of histopathologic and molecular patterns of t-NEPC and AVPC might also facilitate a more uniform nomenclature and classification of these tumors. The identification of more specific and sensitive biomarkers for NED may allow an earlier identification of transdifferentiation in the course of treatment as well as a better monitoring of the applied therapies.

For this aim, functional studies in t-NEPC models need to be combined with recent advances in the field of liquid biopsy – the analysis of tumor cells and tumor cell compounds in body fluids. Liquid biopsies are associated with lower risk for the patients and can be repeatedly applied as required in therapy monitoring, for instance. Importantly, liquid biopsy allows the assessment of intrapatient tumor heterogeneity and, therefore, is a valuable tool to monitor transition stages were adenocarcinoma and t-NEPC cell populations co-exist [159].

Remarkably, the discovery of key aberrations in t-NEPC has already led to the use of directed therapies in clinical trials. For instance, AURKA inhibitor alisertib has been tested in a phase II clinical trial, though the success has been limited to few patients [160].

Due to the high degree of plasticity of AVPC the combination of different treatment approaches may be necessary. Currently, an ongoing phase II clinical trial analyzes the combination of chemotherapy with carboplatin and cabazitaxel with PARP inhibition and anti-PD1 immunotherapy (NCT04592237). More t-NEPC small molecule inhibitors have been analyzed in preclinical trials, but have not yet been tested in the clinic [63, 101, 121].

Summarizing, the transdifferentiation from prostate adenocarcinoma to t-NEPC is a highly complex process mediated by the interplay of various genomic, epigenetic, transcriptional and posttranslational aberrations. Further research is needed to decipher the precise molecular mechanisms driving the disease and to ultimately develop successful therapies.

## Data Availability

Not applicable.

## Abbreviations

ADT: Androgen deprivation therapy
AR: Androgen receptor
AVPC: Aggressive variant prostate cancer
CRPC: Castration resistant prostate cancer
DDR: DNA damage response
EMT: Epithelial-to-mesenchymal transition
ENO2: Neuron-specific enolase 2
H3K4me2/3: Di−/tri-methylated histone H3 lysine 4
H3K9me1/2: Mono−/di-methylated histone H3 lysine 9
H3K27: Lysine 27 in histone 3
H3K27me2: Dimethylated H3K27
KO: Knock-out
mCRPC: Metastatic castration resistant prostate cancer
NE: Neuroendocrine
NEPC: Neuroendocrine prostate cancer
NHA: Novel hormonal agent
NED: Neuroendocrine transdifferentiation
OS: Overall survival
PCa: Prostate cancer
PDX: Patient-derived xenografts
PSA: Prostate specific antigen
TME: Tumor microenvironment
t-NEPC: Treatment-emergent neuroendocrine prostate carcinoma
